# COVID‐19: a pandemic experience that illuminates potential reforms to health research

**DOI:** 10.15252/emmm.202013278

**Published:** 2020-10-19

**Authors:** Hannah N Kozlowski, Michael E Farkouh, Meredith S Irwin, Laszlo G Radvanyi, Aaron D Schimmer, Uri Tabori, Norman D Rosenblum

**Affiliations:** ^1^ Biomedical Engineering University of Toronto Toronto ON Canada; ^2^ MD/PhD Program Temerty Faculty of Medicine University of Toronto Toronto ON Canada; ^3^ Peter Munk Cardiac Centre Heart and Stroke Richard Lewar Centre University of Toronto Toronto ON Canada; ^4^ Department of Paediatrics The Hospital for Sick Children University of Toronto Toronto ON Canada; ^5^ Ontario Institute for Cancer Research Toronto ON Canada; ^6^ Princess Margaret Cancer Centre University of Toronto Toronto ON Canada

**Keywords:** Microbiology, Virology & Host Pathogen Interaction, S&S: Ethics

## Abstract

COVID‐19 has halted research around the globe and forced researchers out of their laboratories. Non‐emergency medical appointments were canceled. Ongoing clinical trials were challenged to create new modes of operation while public pressure mounted to find therapeutic options against COVID‐19. Yet, the inability to conduct research during COVID‐19 was overcome with cooperation, resource sharing, and compassion, which provides important lessons on how to improve health related research as we enter a new normal.

## Graphical abstract

The COVID‐19 crisis necessitates and unprecedented level off cooperation and sharing in biomedical research. It should inspire science policies and funding post‐COVID to help drive forward Open Science.

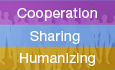

To meet the COVID‐19 challenge, the global scientific community focused human and material resources to develop new drugs, therapies, vaccines, diagnostics, and so on. However, it takes 10–20 years to turn a basic discovery into a new drug via the typical translational path (Paul *et al*, 2010; Mohs & Greig, 2017), which would not be compatible with the goal of rapidly developing diagnostics and therapeutics for COVID‐19. This situation created a need to overcome the many hurdles along the translational research pathway—financing, business development, regulation, product development, commercialization, and different cultures—in order to satisfy an urgent societal need. Here, we discuss the lessons learned from the Toronto Academic Health Sciences Network's (TAHSN) response to COVID‐19. We highlight the critical importance of cooperation and compassion in maintaining productivity during the pandemic and identify opportunities for shaping a new era of research with increased patient engagement, fewer silos, and a shared goal of improving health and decreasing disease burden.

## Themes emerging from the COVID‐19 pandemic

**Figure 1:**
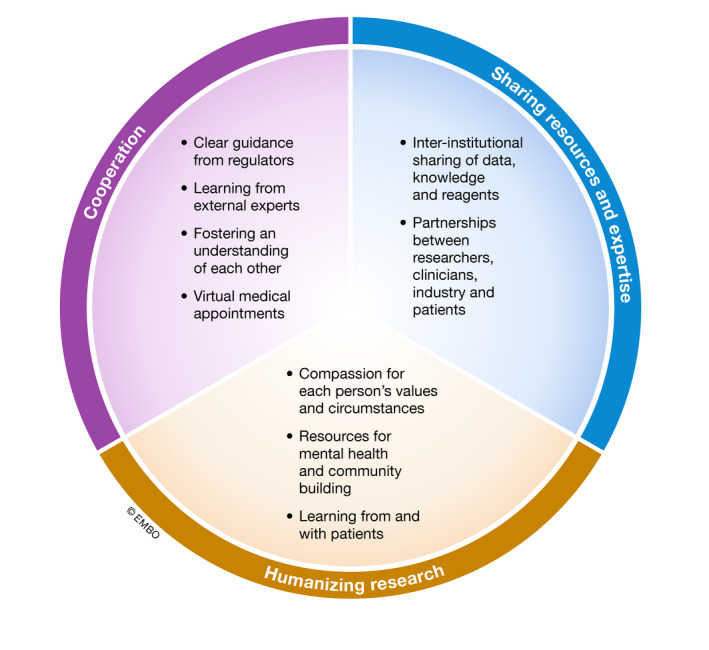
Actions taken by leaders of academic research institutes and departments during COVID‐19 These actions fall into three major themes: cooperation, sharing resources and expertise, and humanizing research. Positive changes in each area are the basis for the creation of impactful research during the “new normal”.

The development of ready‐for‐use clinical products during the COVID‐19 pandemic has engendered cooperativity, engagement, and investment in a common goal (Fig 1), enabled by a shared view of the problem and a genuine desire to contribute to its solution. The fruits of these efforts can be seen around the world as pharmaceutical and biotech companies, and research groups team up to develop, test, and manufacture vaccines (Liu, 2020). This created a real “open science” environment, in which equipment, manufacturing capabilities, patient cohorts, and results were shared in real time (Rouleau, 2020). Prior barriers that obstructed cooperation and sharing were dismantled, facilitating the development of novel ideas into solutions that benefit patients.

### Sharing expertise and resources increased clinical capacity and catalyzed scientific discovery

To expand COVID‐19 testing capacity and reduce redundancy, four medical research institutions in Toronto agreed to a resource‐sharing approach. This is not necessarily new: many philanthropic organizations including the Gates Foundation and the Wellcome Trust have already made open data sharing mandatory for their grants (Kiley *et al*, 2017). In Toronto, this new sharing agreement went further to include samples and reagents to allow free movement between institutes without any attendant intellectual property (IP) agreements.

Two major lessons emerged. First, focusing on the shared goal, rather than first negotiating IP rights, increased productivity. Early conversations around IP are expensive, slow negotiations between institutes and have stymied innovation. Second, sharing resources such as reagents, equipment, staff, and expertise increased the number of problems that could be solved. Without such agreements, hospitals would only test their own patients and the majority of these basic tests indicated only viral load. Instead, cooperation leveraged equipment, protocols, and expertise external to hospitals. The Ontario Institute of Cancer Research, normally a cancer‐focused genomics institute, sequenced indeterminate and positive COVID‐19 samples. They shared the sequences in real time to further the scientific community's understanding of COVID‐19, which exemplifies how resource sharing between institutions can increase testing capacity and support scientific discovery.

New partnerships were built on a foundation of resource‐sharing initiatives and the motivation to contribute during the crisis. In Toronto, new collaborations between academia and industry resulted in multicenter clinical trials that transitioned from idea to approval in as few as 14 days. The scale and speed of these agreements were only possible because academic researchers and private industry worked together with a shared understanding of the problem and jointly identified their collective goals, expertise, available resources, and clinical networks. In one instance, a conversation between two colleagues on the use of immunomodulators to protect patients with cancer from the severe side effects of COVID‐19 infection became a phase three, multicenter clinical trial. The total time from initial conception to protocol drafting to approval by Health Canada was only 6 weeks. We have also seen international pharmaceutical companies share their manufacturing facilities, supply chains, and expertise in scaling (Liu, 2020; The Association of the British Pharmaceutical Industry, 2020). These joint commitments led to further cooperation between researchers, private industry, research ethics boards, and national regulators.

### Cooperation reduced inefficiencies associated with clinical translation

During COVID‐19, regulators worked closely with scientists to quickly translate scientific discoveries into medical interventions. Health Canada released guidance documents for approval of COVID‐19 medical devices, including diagnostic tests, to simplify and streamline the application process, without impacting post‐market safety standards (Reid, 2020). Similarly, the US FDA issued an emergency use authorization that expedited the review of COVID‐19 diagnostics (Reid, 2020). This sort of response by regulators around the world led to increased cooperation with researchers, which boosted productivity.

### Compassion and support for the people behind the research fostered sustained productivity

The research community also realized that supporting research meant supporting each other. Behind each study are researchers with unique values, expectations and experience. Attention to individuals and their circumstances—delays in student graduation, personal comfort in the workplace, safe commuting, family responsibilities, challenges associated with working from home, and uncertainty due to job security—became central to discussions on restarting research after COVID‐19 restrictions are loosened. For example, flexible working times and shift work allowed for physical distancing, avoiding rush hours, and benefited families with working parents. Beyond any particular research program and institution, TAHSN provided virtual resources, seminars, social interactive space, and training to promote support research staff and foster a positive working environment, all of which strengthened the research community and sustained productivity.

During the pandemic, several non‐COVID‐19 trials were also forced to adapt. Running these trials without compromising rigor or the safety of patients required cooperation between researchers, local research ethics boards (REBs), patients, and patient advocates to address patient anxieties and clinical priorities by developing new protocols. In Toronto and medical clinics worldwide, clinicians adapted by moving medical visits and clinical trial appointments online. Using video calling, physicians saw their patients in their home environment as well as other family members who joined the call. Although these interactions provided a broader view of the patient, their health, and their environment, careful retrospective analyses of the safety, reliability, and effectiveness of virtual patient assessments are still needed to determine if this approach is sustainable.

## A framework for the “new normal”

Harnessing lessons learned during COVID‐19 to improve health research requires joint action to further improve research infrastructure, integrate parts of the translational pathway, and support the people who do the work. Here, we build upon those lessons to propose a framework for the “new normal” after the first phase of COVID‐19 (Table 1). This includes creating infrastructures that promote cooperation, support team science, and prioritize the wellness of people. The proposed framework can help to break down research silos and efficiently move scientific discoveries from the laboratory to the clinic.

**Table 1:** Summary of changes implemented during COVID‐19 that benefited the advancement of medical discoveries. Further benefits can be seen if long‐term recommendations are implemented

Themes	Short‐term changes to adjust for the impact of COVID‐19	Long‐term changes inspired by the changing COVID‐19 environment
Sharing resources and expertise	Created new data‐sharing agreements without extended IP negotiations	Keep focused on producing research that benefits patients
	Formed partnerships between researchers, clinicians, industry, and patients to accelerate the development of solutions to current problems	Organize grand rounds so that clinicians and scientists from different disciplines give joint talks on the same problem
Cooperation	Regulators released clear guidance documents to streamline regulatory approval applications	Create research clusters that promote sharing and cooperation between basic science, health service, implementation, engineering, and clinical researchers; patient advocates and private industry
	REB members learned from non‐REB scientists to efficiently and thoroughly review ethics applications	Recognize researchers for collaborative or middle author roles in order to promote team science
	REBs, researchers and patients worked together to revise clinical trial protocols	Reward non‐publication achievements (i.e., patient enrollment, clinical guidelines)
	Clinicians and researchers conducted medical appointments over the phone or online	Create government‐industry grants that support the long‐term development of scientific discoveries into clinical impact
		Introduce young scientists to alternate career paths (i.e., entrepreneurship)
Humanizing research	Acknowledged and discussed researchers’, staff’, and students’ unique values and circumstances	Continue thinking about health and personal circumstances of all people involved in research
	Increased resources for mental health and community building	Continue virtual and in‐person resource sharing to promote good mental health and foster community
	Enhanced researcher consultation with patients to understand their disease, their experience, and what matters to them	Continue to engage patients from inception and throughout a project
		Develop pathways for students to learn from patients and experience medical clinics

To *foster cooperation,* we need to assemble team members of the broader research and translation community around a shared goal. During COVID‐19, researchers, industry professionals, and regulators were each determined to find solutions that benefit patients right away. Maintaining this shared focus going forward requires that we rebuild activities to support cooperation. In Toronto, we started by restructuring hospital grand rounds, the institute‐wide clinical seminars, to educate the community on a relevant medical topic. Clinicians and researchers from different fields now give joint talks to highlight the larger problem and possible solutions from their perspective. This was particularly helpful during COVID‐19 as infectious‐disease clinicians discussed the newfound pediatric presentation of COVID‐19 and immunologists explained the immune response and how that informs vaccine development. Moving forward, collaborative research should become a model for trainees and other researchers to strive for as they see the fruitful outputs of teamwork.

To further *support cooperation* and build partnerships, we need to invest in research clusters that are grouped by disease or clinical question instead of methods. Each cluster would include basic science, health services, implementation, engineering, and clinical expertise. Seminars within each cluster would describe different facets of the problem, as per the expertise of the speaker. This provides researchers and trainees a shared view of the big picture so that they can readily come together to solve the problem. These collaborative clusters should also include patient advocates and industry to open communication and help different groups to understand each other and facilitate a shared culture. In such a disease‐focused research cluster, the role of each member will be clearly understood, reducing the number of barriers to producing research that benefits patients.

To *encourage resource sharing* and to move research discoveries closer to patients, research institutions need to value and reward teamwork. Yet, the current academic structure does not always reward team science or the building of collaborative networks. In the medium‐term, we should change how we evaluate scientific contributions and create promotional metrics that recognize team science. In Toronto, we acknowledge that success may not always result in a publication but in the adoption of a new policy, better clinical guidelines, a new partnership with industry or the clinical validation and implementation of a diagnostic test. In the long term, we should provide more grants that promote collaboration akin to the European Union's Research Programmes that provide long‐term funding for collaborations between multinational research groups.

Supporting long‐term development relieves the pressure to publish quickly and often and encourages teams to tackle big problems. Given the time and resource needed to bring new interventions to the clinic, the funds for these grants should come from government‐industry partnerships, and there should be an option of applying for additional funding if pre‐specified milestones are met (van Dijk *et al*, 2019).

Producing research that is meaningful to patients also requires that we *humanize research*. During COVID‐19, there was a shift from thinking about research as something that eventually trickles down to a “patient”, to understanding that we all may benefit. This makes it more natural to think of patients as the reason and focus of our scientific inquiries. In Toronto, we previously started shifting toward patient‐centered research by involving patients and families in advisory committees, ethics boards, and research teams as charitable foundations have been doing for years (Stevens, 2019).

We can further strengthen this partnership by creating pathways for students, the researchers‐of‐tomorrow, to learn from patients directly. In these pathways, students will spend more time in the clinic, meet patients and caregivers, write REB proposals, analyze the flow of information between health professionals, and observe medical procedures (DelNero & McGregor, 2017). For example, asking patients why they continue/discontinue their treatments and what quality of life indicators/outcomes matter to them can inform study design and give students a sense that they are contributing to improve patient health. This experience also breaks down barriers between research and clinic. For researchers, it provides a broader understanding of a disease and the challenges of implementation. For patients and caregivers, it is an opportunity to share their wisdom and learn about the research process.

## Change should happen now

Now is the time to harness the lessons learned from COVID‐19 and shape a new era of innovation that more effectively brings the benefits of research to patients. We cannot let the positive changes be lost. First, we need to continue to create joint goals between all members of the translational path. Sharing must continue to extend beyond reagents and funding. These actions can be strengthened by restructuring our institutions to reinforce “open science”, developing broad data‐sharing agreements and creating collaborative work spaces. Second, we need to value and reward teamwork. This involves creating new measures of academic success and collaborative grants. The validation of new diagnostic tests, the implementation of a new workflow or pharmacokinetic studies for a new treatment is not considered high‐impact science, but it is needed to improve quality of life and clinical practice. Third, funding must be shared between immediate clinical need and continuous support for basic science to enable the biological understanding of disease. The rapid development of diagnostics and vaccines for COVID‐19 was based on years of basic research in virology, therapeutics, and engineering. Lastly, we must remember that people are at the heart of research. We must work to make sure all team members are supported and that patients are engaged in research. Together, we can create a new era of medical research, where knowledge moves fluently between patients, researchers, industry, regulators, and policy makers in order to generate solutions that are used in the clinic.

## Author contributions

H.N.K. and N.D.R. conceived the idea, wrote and edited the manuscript; all authors contributed content and reviewed the final manuscript.

## Conflict of interest

ADS has received research funding from Takeda Pharmaceuticals and Medivir AB and consulting fees/honorarium from Novartis, Jazz, and Otsuka Pharmaceuticals. ADS also holds stock in Abbvie. UT, LR, MF, MI, HK, and NDR have no relevant relationships to disclose.
